# Case Report: Giant abdominal hemangioma originating from the liver

**DOI:** 10.3389/fonc.2023.1165195

**Published:** 2023-07-31

**Authors:** Tianxiang Jiang, Zhou Zhao, Zhaolun Cai, Chaoyong Shen, Bo Zhang

**Affiliations:** ^1^ Department of General Surgery, West China Hospital, Sichuan University, Chengdu, China; ^2^ Gastric Cancer Center, West China Hospital, Sichuan University, Chengdu, China

**Keywords:** giant, hemangioma, pedunculated, liver, abdominal

## Abstract

**Background:**

Hepatic hemangioma is among the most common benign liver lesions. However, giant pedunculated hepatic hemangiomas are exceptionally rare and associated with additional risks, such as torsion.

**Case presentation:**

We present the case of a 63-year-old female patient who presented with abdominal distension and pain. Barium meal examination and gastroscopy revealed a large, smooth-surfaced submucosal bulge located at the fundus of the stomach. Subsequent MRI examination identified a mass measuring approximately 6.4 x 7 cm in the left upper abdomen. Surgical intervention was planned for mass removal. However, intraoperative exploration revealed the origin of the mass to be the liver, and subsequent histopathological examination confirmed it as a hemangioma.

**Conclusion:**

We systematically summarized the characteristics of our case along with 31 previously reported cases. Giant pedunculated hepatic hemangiomas typically occur in the left lobe of the liver. Due to their atypical presentation, a combination of imaging methods such as ultrasound, CT, and/or MRI is essential for accurate diagnosis. Furthermore, surgical intervention is recommended due to the potential risks of bleeding, rupture, and torsion.

## 1 Introduction

Hemangiomas are benign tumors that may occur anywhere in the abdomen, including solid organs, hollow viscera, ligaments, and abdominal wall, of which liver is the most common site (1). Hepatic hemangiomas are a prevalent type of benign liver lesion, second only to focal fat sparing and liver cysts in frequency (2, 3). The prevalence of hepatic hemangiomas detected through radiological imaging ranges from approximately 2.5% to 3.3% (3, 4). Typically, hepatic hemangiomas do not cause symptoms; however, when they reach a significant size, they may give rise to non-specific gastrointestinal manifestations (5). While imaging studies often aid in diagnosing hepatic hemangiomas, their presentation can occasionally deviate from the norm, leading to confusion with malignant lesions (6, 7). Although hepatic hemangiomas primarily occur within the liver, instances of giant pedunculated hemangiomas outside the liver capsule are exceedingly rare (8).

This report describes the case of a 63-year-old female patient who presented with a gastric submucosal tumor detected during gastroscopy. Subsequent MRI imaging revealed the presence of a large tumor located between the stomach and spleen within the abdominal cavity. Due to suspicion of a giant abdominal tumor, the patient underwent surgical intervention. However, during surgery, it was found that the tumor was a pedunculated tumor of the liver. The histopathological examination of the specimen revealed a hemangioma. This case has been documented in accordance with the CARE Guidelines (**Supplementary Table S1**).

## 2 Case description

A 63-year-old female patient was admitted to our hospital for examination and treatment on July 26, 2022. Six months prior to admission, she experienced upper and middle abdominal distension after eating, which was slightly relieved by anal venting. She reduced the amount of food consumed per meal and did not experience any further bloating. One month prior to admission, she developed vague right upper abdominal pain after consuming fatty foods, which was paroxysmal and tolerable and improved with a light diet. No other symptoms such as acidity, eructation, vomiting, or diarrhea were reported.

**Figure 1:**
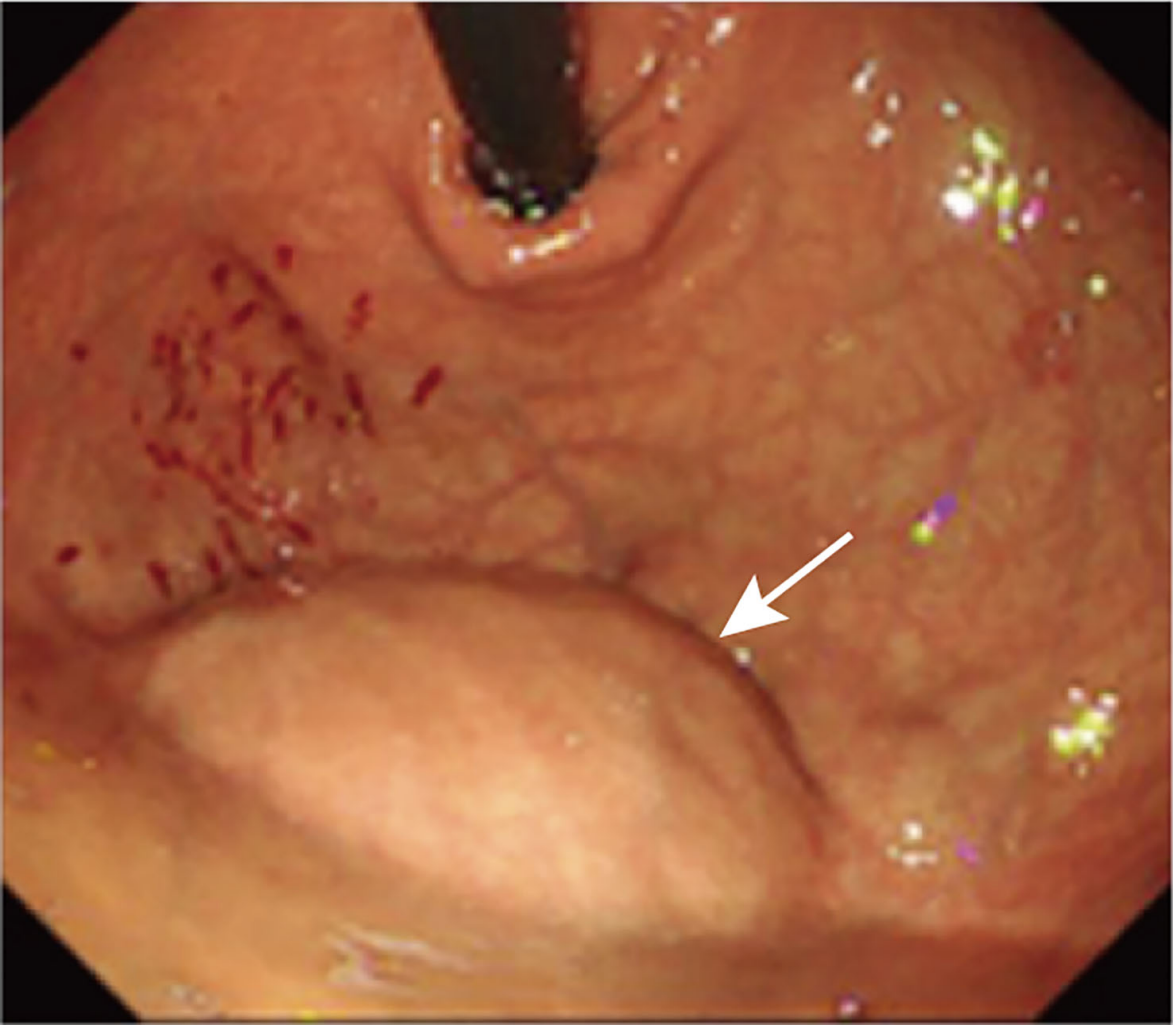
Endoscopy.

**Figure 2:**
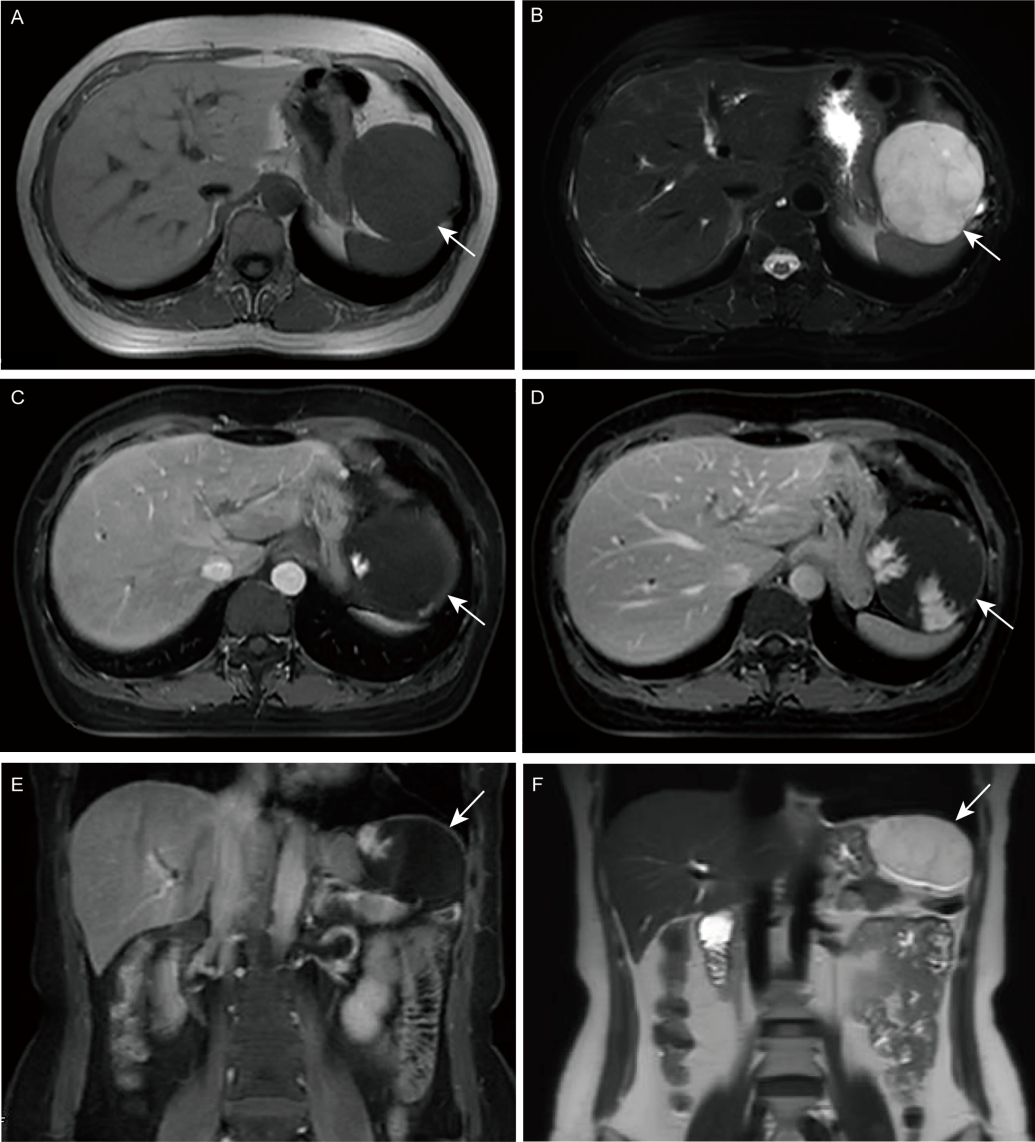
Magnetic resonance imaging (MRI). **(A, E)** T1-weighted image (T1WI); **(B, F)** T2-weighted image (T2WI); **(C)** Enhanced arterial phase; **(D)** Enhanced portal phase.

Upon initial physical examination, pain was present in the left upper abdomen upon pressure, without rebound pain. The rest of the examination was unremarkable. Further investigation through barium meal examination, endoscopy, and abdominal MRI was arranged. Barium meal examination revealed a locally depressed fundus of the stomach with a smooth mucosal surface (**Supplementary Figure S1**), while endoscopy showed a large submucosal bulge with a smooth surface at the fundus of the stomach (**Figure 1**), suspected to be gastric submucosal tumor. MRI detected a 6.4 x 7 cm mass in the left upper abdomen with low signal on T1-weighted images and high signal on T2-weighted images, with partial intensification in the arterial phase and progressive intensification in the venous phase (**Figure 2**).

**Figure 3:**
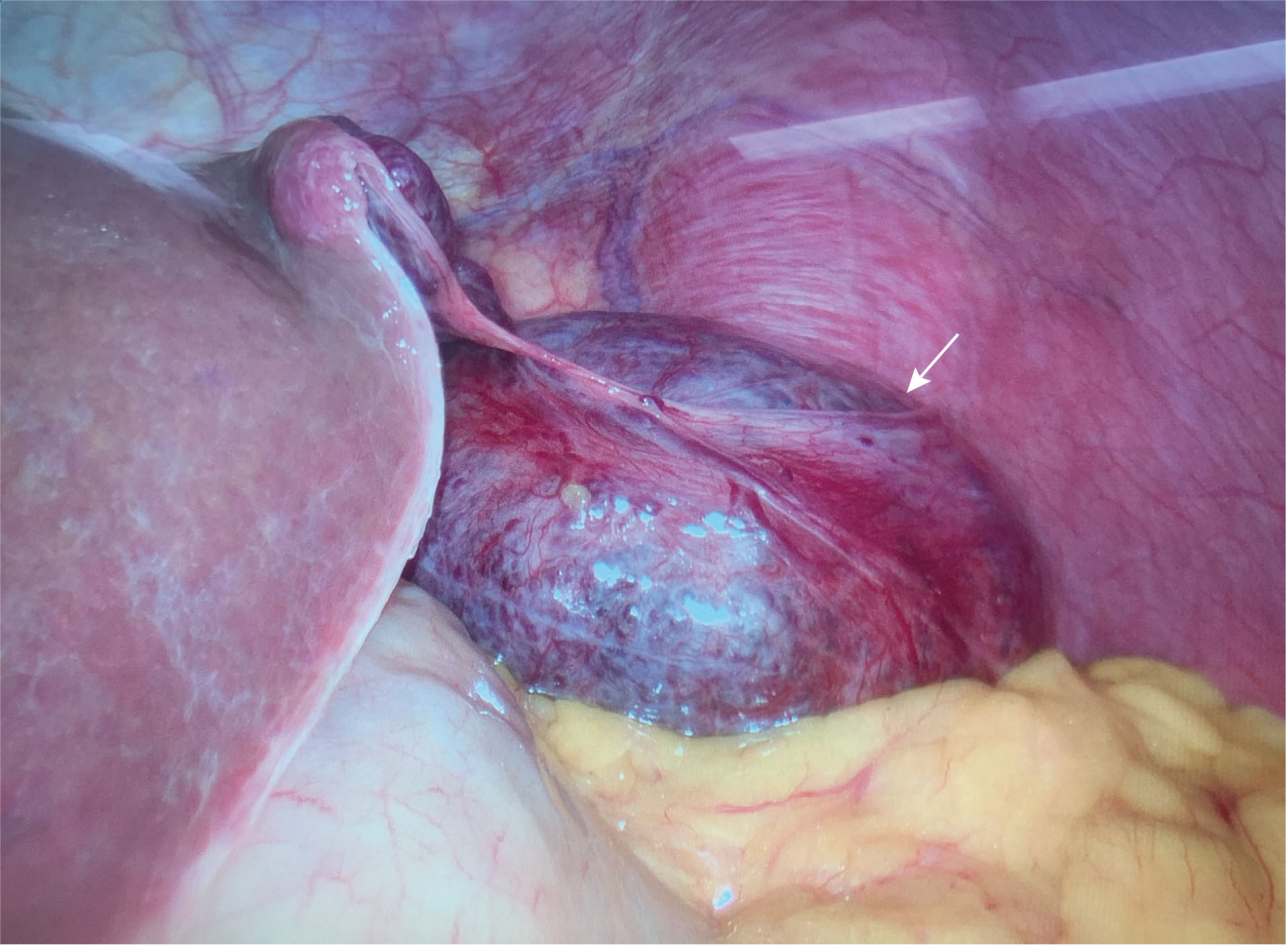
Intraoperative photograph.

**Figure 4:**
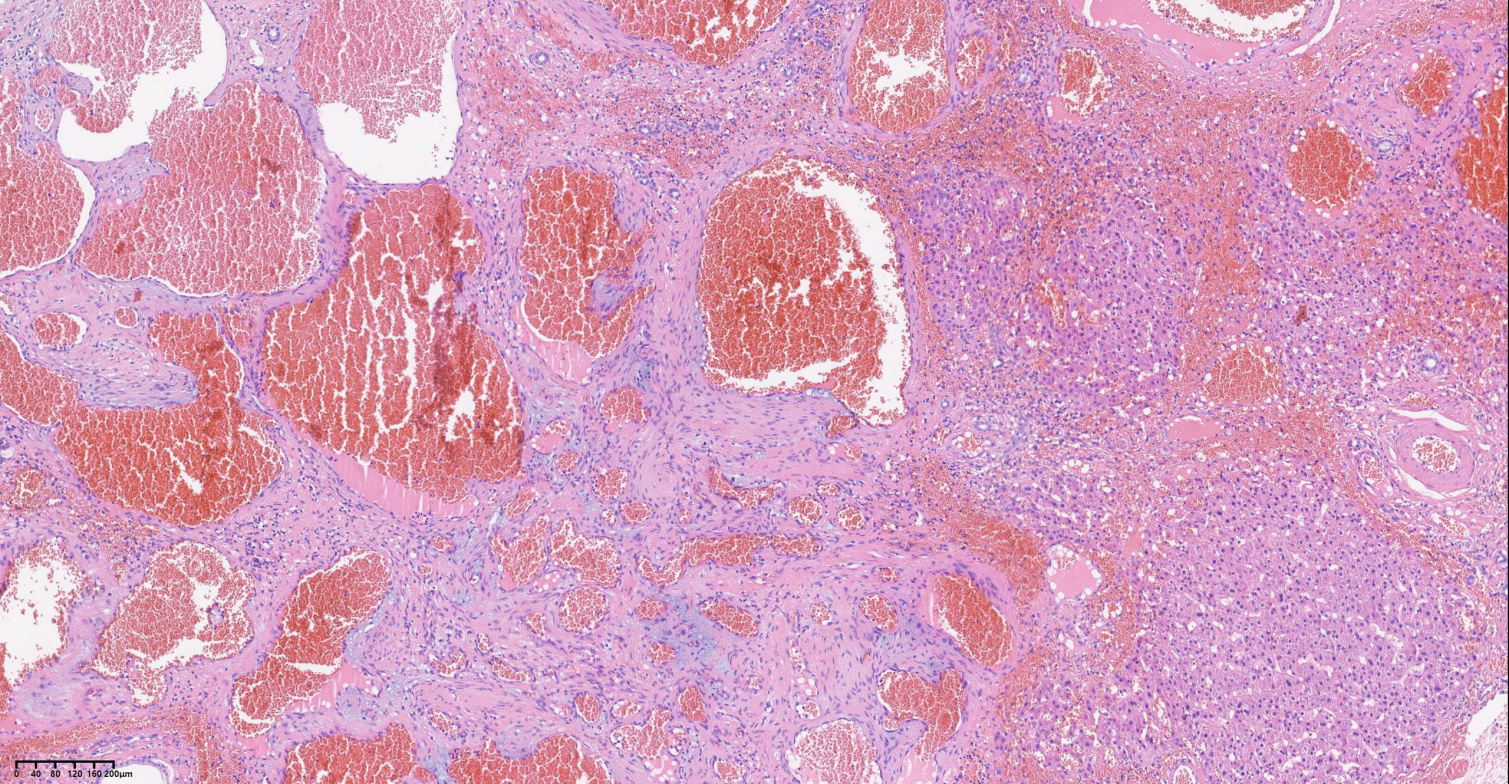
Post-operative histopathological slides of the mass with hematoxylin and eosin staining.

Laparoscopic surgery was initially planned; however, intraoperatively, it was discovered that the mass was connected to the liver by pedunculi. The mass exhibited dimensions of 9 * 8 cm and had a soft consistency with angulated surface vessels closely associated with the lesser curvature of the stomach (**Figure 3**). The mass was removed en bloc with 2 cm margins. The final pathology report indicated a hemangioma (**Figure 4**). The patient timeline of diagnostic testing and Therapeutic intervention are shown in **Supplementary Figure S2**. A follow-up telephone call conducted three months post-operation revealed that the patient had experienced resolution of symptoms and reported no additional discomfort.

## 3 Patient perspective

Prior to my admission to West China Hospital of Sichuan University, I was plagued by symptoms that restricted my dietary intake, significantly diminishing my quality of life. I was apprehensive about the prospect of undergoing a partial gastrectomy, as it could potentially impact my future dietary habits. However, I was pleased to discover that my fears were unfounded, as my symptoms showed a marked improvement after being treated at West China Hospital of Sichuan University. This resulted in a significant enhancement of my overall quality of life.

## 4 Discussion

Hemangioma is a benign tumor that is commonly asymptomatic when its size is less than 5 cm. Larger hemangioma may present symptoms such as abdominal pain, discomfort, and a sensation of fullness in the abdomen (9). Imaging studies, such as ultrasound, CT scans, and MRI, can aid in the diagnosis of hemangioma (10). MRI scans typically depict hemangioma as well-defined, smooth, homogenous lesions that are hypointense on T1-weighted images and hyperintense on T2-weighted images (5). Fine-needle aspiration biopsy(FNAB) is a common diagnostic tool for tumors, however, this method carries a risk of hemorrhage due to the vascular-rich nature of hemangioma (11). A study conducted by Heilo et al. found that ultrasound-guided FNAB had a false-negative rate of 15-16 out of 51 patients with suspected hepatic hemangioma (12). Several studies have suggested that the low diagnostic rate and the risk of bleeding associated with FNAB make it an unsuitable method for diagnosing hepatic hemangioma (10, 13). It is important to note that hepatic hemangioma can sometimes be mistaken for other liver diseases, such as hepatocellular adenoma, focal nodular hyperplasia, or hepatocellular carcinoma. However, cases where hepatic hemangioma mimics GIST are extremely rare.

For cases of gastric fundic masses of unknown origin, adequate investigations are required to assist in the diagnosis. Besides routine CT, MRI and endoscopy, it is necessary to perform ultrasonography (14) and ultrasound endoscopy (15) to clarify the nature of the mass and its relationship to the surrounding organs. FNAB is a valuable diagnostic tool for various types of tumors, particularly for biopsying superficial or palpable lesions in organs such as the thyroid, breast, lymph nodes, and others (16–22). While FNAB is considered a safe and effective method, it has a potential risk for bleeding and false negative results. Therefore, it may not be appropriate for all patients, particularly in cases of hepatic hemangioma (11, 23). To enhance the safety of FNAB, ultrasound guidance is recommended (24).

While hepatic hemangiomas generally exhibit a slow growth rate (25), the presence of large hepatic hemangiomas increases the risk of bleeding and rupture (4). Hemangiomas exceeding 4 cm in diameter are categorized as giant hemangiomas (26). Moreover, in the case of large, pedunculated hepatic hemangiomas, there is a potential risk of torsion, which can result in acute abdominal pain and pose a life-threatening situation (27). To date, only a limited number of cases involving giant pedunculated hepatic hemangiomas have been reported.

A systematic search was conducted in the PubMed and Embase databases to identify relevant studies. After removing duplicates, a total of 37 studies were retrieved. The search strategy employed was (((hepatic) OR (liver)) AND (hemangioma)) AND (pedunculated). Through careful evaluation of full-text articles and exclusion of studies with irrelevant topics, tumors smaller than 4 cm, and those lacking full-text availability, 19 studies remained. Furthermore, 9 additional studies were retrieved from the references of the included studies. In total, 28 studies (8, 27–52) encompassing 31 cases of giant pedunculated hepatic hemangioma were identified. A summary of the characteristics of these previous cases, as well as our own case, is provided in **Table 1**. The incidence of giant pedunculated hepatic hemangioma was found to be higher in females (n=22) compared to males (n=10). The median age at diagnosis was 65 years, with a range of 26 to 71 years. The left lobe of the liver was the most common location for these tumors. Out of the cases, 11 (34.4%) were asymptomatic, and 5 cases were initially suspected to be gastrointestinal interstitial tumors prior to surgery (27, 29, 41, 43, 44). Surgical resection was the predominant treatment approach in almost all cases.

**Table 1:** Cases of giant pedunculated hepatic hemangiomas in the literature.

Study	Sex	Age	Symptoms	Liver lobe	Size*	Radiology	Treatment
Ellis JV, et al. (30)	M	67	No	Right	11	US, CT, Arteriography	Surgery
Srivastava DN, et al. (33)	F	53	Abdominal mass, pain	Left	NA	CT, MRI, Arteriography	Angiographic embolization, surgery
Nishiyama Y, et al. (32)	F	55	Abdominal pain and discomfort	Left	NA	CT, Blood-pool scintigraphy, SPECT	NA
Tsai CC, et al. (29)	F	53	No	Left	15	CT, SPECT	NA
Cortes-Blanco A, et al. (28)	F	60	Jaundice	Left	NA	US, CT, Labeled erythrocyte scintigraphy	Surgery
Liang RJ, et al. (31)-1	M	42	No	Left	5	US, CT, MRI, Labeled erythrocyte scintigraphy, Arteriography	Surgery
Liang RJ, et al. (31)-2	M	36	No	Left	5	US, CT, MRI, Arteriography	Followed up
Guenot C, et al. (39)	F	48	Abdominal pain, heaviness in the abdomen	Left	12	US, CT	Surgery
Kudara N, et al. (42)	M	41	No	Left	6.5	Barium meal examination, EUS, CT, MRI, Arteriography	Surgery
Kukuk GM, et al. (37)	F	58	Abdominal pain, vomiting	Left	6	Endoscopy, Barium meal examination, CT, US	Surgery
Vivarelli M, et al. (36)	M	45	Abdominal pain, nausea, low-grade fever	Left	NA	CT	Surgery
Moon HK, et al. (43)	F	56	Abdominal discomfort, dyspepsia	Left	4.5	Barium meal examination, CT	Surgery
Darzi A, et al. (27)	F	45	Abdominal pain, nausea, vomiting, loss of appetite	Left	10.5	X-ray, US, CT, endoscopy	Surgery
Hajjam ME, et al. (34)	F	66	No	Left	7.5	CT, MRI	Surgery
Melfa G, et al. (8)	F	62	Dyspepsia	Left	8	CT, MRI	Surgery
Al Farai A, et al. (41)	M	48	Diarrhea, vomiting and abdominal pain	Left	20	US, CT, MRI	Surgery
Kouki S, et al. (35)	F	26	Abdominal pain	Left	9	US, MRI	NA
Krishnan V, et al. (44)	F	51	No	Left	11.2	CT, MRI, US	Surgery
Castañeda Puicón L, et al. (38)	M	26	Abdominal pain, mass	Left	13	CT	Surgery
Bouknani N, et al. (40)	F	59	Abdominal pain	Right	4	CT	Surgery
Parikh VP, et al. (46)	M	71	No	Left	NA	CT	Surgery
Tran-Minh VA, et al. (45)	F	47	Abdominal pain	Left	8.5	US, CT	Surgery
Cortes-Blanco A, et al. (28)	F	60	Jaundice	Left	15	US, CT, Labeled erythrocyte scintigraphy	Surgery
Needleman L, et al. (47)	F	NA	No	Left	NA	US, CT	Surgery
Liessi G, et al. (48)-1	M	58	Abdominal pain	Right	4	US, CT	Surgery
Liessi G, et al. (48)-2	M	48	No	Right	4	US, CT	NA
Liessi G, et al. (48)-3	F	45	No	Right	4	US, CT	NA
Ersoz F, et al. (49)	F	31	Abdominal pain	Right	15	CT	Surgery
Karatzas T, et al. (50)	F	63	Acute intestinal obstruction	Right	14	CT, MRI	Surgery
Acharya M, et al. (51)	F	37	Abdominal pain	Left	10	CT	Surgery
Candido Pde C, et al. (52)	F	28	Abdominal mass, discomfort	Right	18	US, CT, MRI	Surgery
Our case	F	63	Abdominal distension, bloating	Left	9	Barium meal examination, endoscopy, MRI	Surgery

M, Male; F, Female; NA, not available; *size is indicated in centimeters.

In this report, we present a case of a 63-year-old female patient who was diagnosed with a large hemangioma located interposed the spleen and the stomach. The hemangioma measured 9 * 8 cm in size and was initially mistaken as a GIST based on endoscopy showed that the tumor was a spherical submucosal bulge from the fundus of the stomach (53). Although FNAB is typically a safe procedure (54, 55), it was not performed in this case due to the potential risk of hemorrhage. Given the significant size of the mass, the presence of symptoms and the risk of torsion, surgical resection was performed. During the procedure, it was revealed that the tumor originated from the liver. Pathological analysis postoperatively confirmed the diagnosis of a hemangioma.

## 5 Conclusion

Giant pedunculated hepatic hemangioma is a rare benign tumor primarily originated from the left lobe of the liver. The symptoms associated with this condition are typically non-specific or even asymptomatic, leading to potential confusion with various other upper abdominal masses. Accurate diagnosis of this atypical hemangioma requires a comprehensive approach utilizing multiple imaging techniques, including ultrasound, CT, and/or MRI. Considering the risks of bleeding, rupture, and torsion, surgical intervention is the recommended treatment modality for large pedunculated hepatic hemangiomas.

## Data availability statement

The original contributions presented in the study are included in the article/**Supplementary Material**. Further inquiries can be directed to the corresponding author.

## Ethics statement

Written informed consent was obtained from the participant/patient(s) for the publication of this case report.

## Author contributions

TJ – manuscript write up, data collection, and patient interview. ZZ – manuscript write up, data collection, and patient interview. ZC – manuscript write up and literature review. CS – surgery perform. BZ – surgery perform. TJ and ZZ provided equal contributions and shared co-first authorship. All authors contributed to the article and approved the submitted version.

## Supplementary material

Supplementary Figure S1 Barium meal examination.

Supplementary Figure S2 Timeline.

Supplementary Table S1 CARE-checklist.
