# Supplementary figures and images for: Case Report: Giant abdominal hemangioma originating from the liver
Source: Front Oncol. 2023 Jul 31;13:1165195. doi: 10.3389/fonc.2023.1165195 (PMC10425808; doi:10.3389/fonc.2023.1165195)

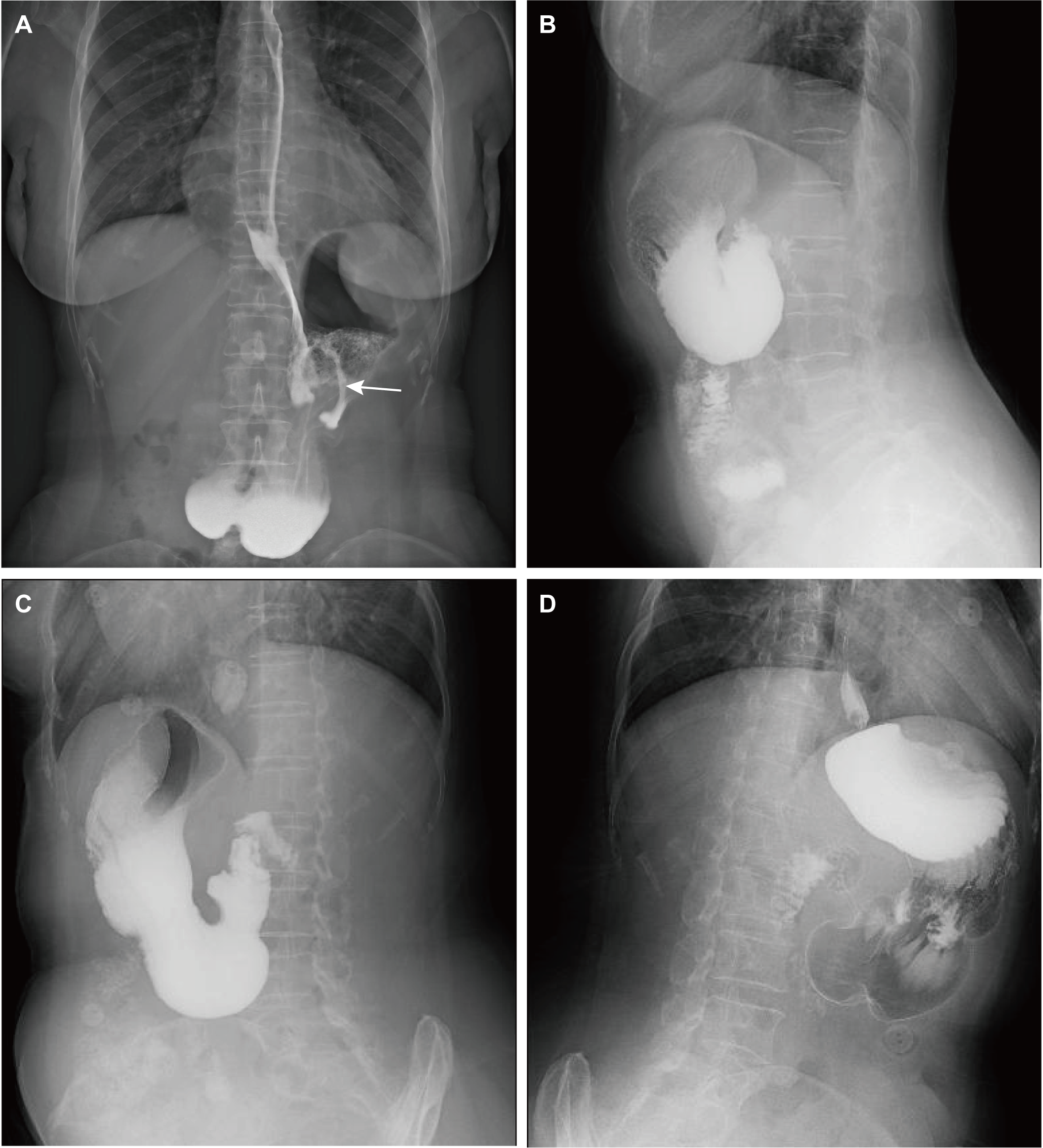

Supplement: Supplementary Figure S1 — Barium meal examination. [file Image_1.tif]

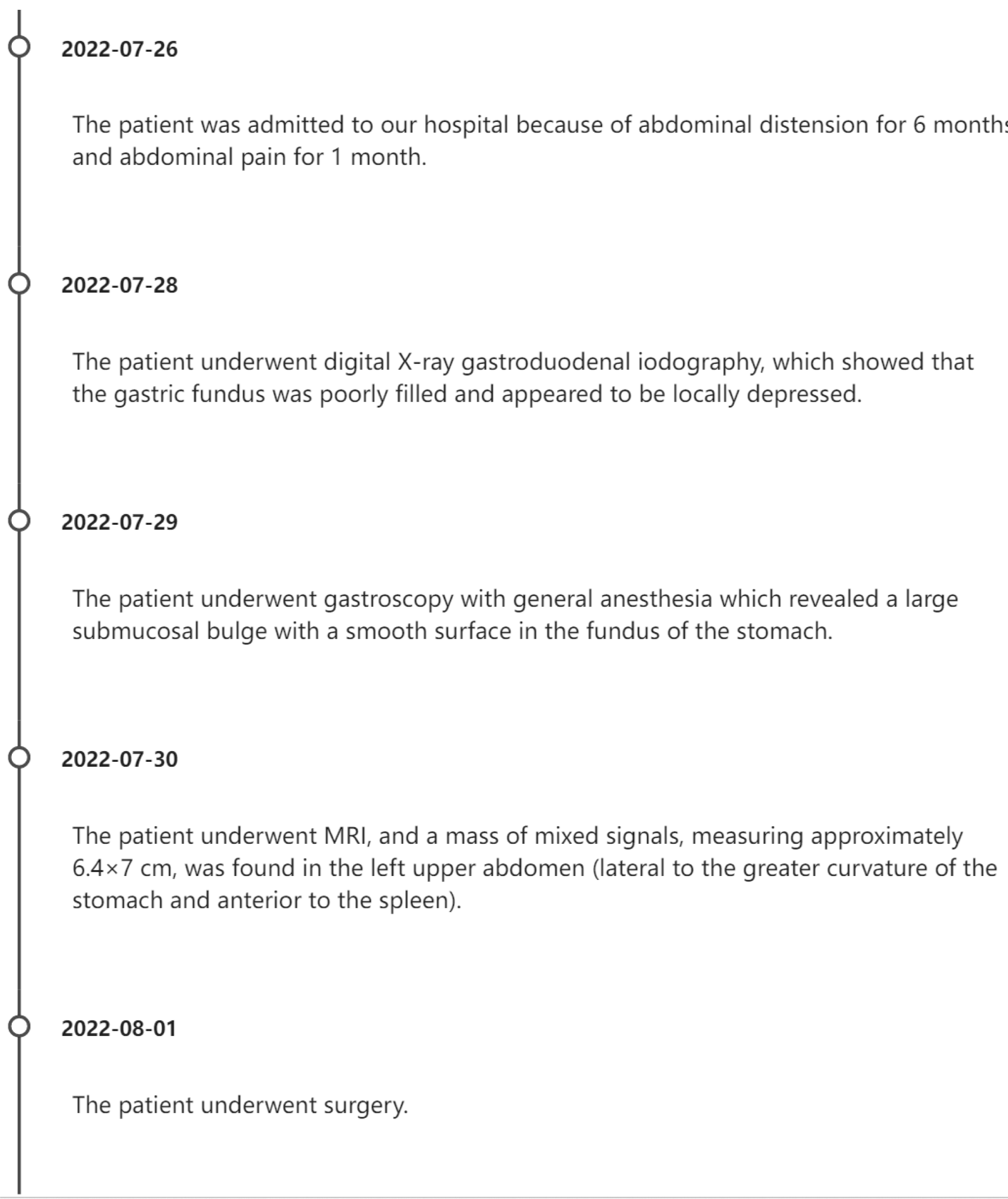

Supplement: Supplementary Figure S2 — Timeline. [file Image_2.tif]
