# Supplementary material for: Case Report: Giant abdominal hemangioma originating from the liver
Source: Front Oncol. 2023 Jul 31;13:1165195. doi: 10.3389/fonc.2023.1165195 (PMC10425808; doi:10.3389/fonc.2023.1165195)
Supplement: Supplementary Table S1 — CARE-checklist. [file DataSheet_1.docx]

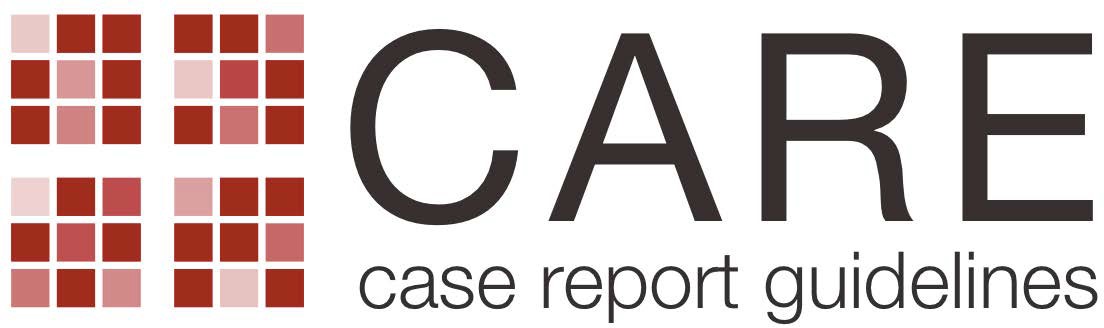
**CARE Checklist of information to include when writing a case report
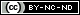
**

**Topic Item Checklist item description Reported on Line**

**Title 1** The diagnosis or intervention of primary focus followed by the words “case report” Line 1

**Key Words 2** 2 to 5 key words that identify diagnoses or interventions in this case report, including "case report" Line 7-8

# Abstract

**(no references)**

**3a** Introduction: What is unique about this case and what does it add to the scientific literature? Line 17-18

**3b** Main symptoms and/or important clinical findings Line 11-14

**3c** The main diagnoses, therapeutic interventions, and outcomes Line 14-17

**3d** Conclusion—What is the main “take-away” lesson(s) from this case? Line 17-18

**Introduction 4** One or two paragraphs summarizing why this case is unique (**may include references**) Line 20-31

**Patient Information 5a** De-identified patient specific information Line 33-34

**5b** Primary concerns and symptoms of the patient Line 34-39

**5c** Medical, family, and psycho-social history including relevant genetic information NA

**5d** Relevant past interventions with outcomes NA

# Clinical Findings

**Timeline**

**Diagnostic Assessment**

**Therapeutic Intervention**

**Follow-up and Outcomes**

1. Describe significant physical examination (PE) and important clinical findings Line 40-41
2. Historical and current information from this episode of care organized as a timeline Line 52-53

**8a** Diagnostic testing (such as PE, laboratory testing, imaging, surveys). Line 41-47

**8b** Diagnostic challenges (such as access to testing, financial, or cultural) NA

**8c** Diagnosis (including other diagnoses considered) Line 51-52

**8d** Prognosis (such as staging in oncology) where applicable Line 53-55

**9a** Types of therapeutic intervention (such as pharmacologic, surgical, preventive, self-care) Line 48

**9b** Administration of therapeutic intervention (such as dosage, strength, duration) NA

**9c** Changes in therapeutic intervention (with rationale) NA

**10a** Clinician and patient-assessed outcomes (if available) Line 53-54

**10b** Important follow-up diagnostic and other test results Line 51-52

**10c** Intervention adherence and tolerability (How was this assessed?) NA

**10d** Adverse and unanticipated events NA

**Discussion 11a** A scientific discussion of the strengths AND limitations associated with this case report Line 95-103

**11b** Discussion of the relevant medical literature **with references** Line 63-94

**11c** The scientific rationale for any conclusions (including assessment of possible causes) Line 101-103

**11d** The primary “take-away” lessons of this case report (without references) in a one paragraph conclusion Line 105-108

**Patient Perspective 12** The patient should share their perspective in one to two paragraphs on the treatment(s) they received Line 56-62

**Informed Consent 13** Did the patient give informed consent? Please provide if requested . . . . . . . . . . . . . . . . . . . . . . . . . . . . . . . . . . . . . . **Yes No**
